# Tat Peptide-Mediated Soluble Expression of the Membrane Protein LSECtin-CRD in *Escherichia coli*

**DOI:** 10.1371/journal.pone.0083579

**Published:** 2013-12-16

**Authors:** Guofu Dong, Changzhen Wang, Yonghong Wu, Jianbo Cong, Li Cheng, Mingqun Wang, Pengkai Zhao, Li Tang, Chenggang Zhang, Ke Wu

**Affiliations:** 1 Beijing Institute of Radiation Medicine, Beijing, P. R. China; 2 Beijing Institute of Radiation Medicine, State Key Laboratory of Proteomics, Cognitive and Mental Health Research Center of PLA, Beijing, P. R. China; 3 Beijing Institute of Radiation Medicine, Department of Genomics and Proteomics, Chinese Human Genome Center, Beijing, P. R. China; Louisiana State University and A & M College, United States of America

## Abstract

The human liver and lymph node sinusoidal endothelial cell C-type lectin (hLSECtin), a type II integral membrane protein, containing a Ca^2+^-dependent carbohydrate recognition domain (CRD), has a well-established biological activity, yet its three-dimensional structure is unknown due to low expression yields and aggregation into inclusion bodies. Previous study has demonstrated that the HIV-1 virus-encoded Tat peptide (‘YGRKKRRQRRR’) can increase the yields and the solubility of heterologous proteins. However, whether the Tat peptide could promote the high-yield and soluble expression of membrane proteins in *Escherichia coli* is not known. Therefore, the prokaryotic expression vector pET28b-Tat-hLSECtin-CRD (using pET28b and pET28b-hLSECtin-CRD as controls) was constructed, and transformed into *E. coli* BL21 (DE3) cells and induced with isopropyl-β-d-thiogalactoside (IPTG) followed with identifying by SDS-PAGE and Western blot. Subsequently, the bacterial subcellular structure, in which overexpressed the heterologous proteins Tat-hLSECtin-CRD and Tat-free hLSECtin-CRD, was analyzed by transmission electron microscope (TEM) respectively, and the mannose-binding activity of Tat-hLSECtin-CRD was also determined. Expectedly, the solubility of Tat-LSECtin-CRD significantly increased compared to Tat-free LSECtin-CRD (**p < 0.01) with prolonged time, and the Tat-LSECtin-CRD had a significant mannose-binding activity. The subcellular structure analysis indicated that the bacterial cells overexpressed Tat-hLSECtin-CRD exhibited denser region compared with controls, while dot denser region aggregated in the two ends of bacterial cells overexpressed Tat-free hLSECtin-CRD. This study provided a novel method for improving the soluble expression of membrane proteins in prokaryotic systems by fusion with the Tat peptide, which may be potentially expanded to the expression of other membrane proteins.

## Introduction

Membrane proteins often act as cellular receptors, transporters and various ion channels, and are involved in several crucial cellular processes, such as immune responses, nutrient uptake and nervous system signaling [1-6]. A structural understanding of these membrane proteins is beneficial to elucidating the role that each plays in the above processes [7,8]. However, there are several limitations to the study of membrane proteins structure, such as low-yield expression and localization in inclusion bodies [9,10]. To date, the high-yield expression of soluble, functional membrane proteins for structural studies has been notoriously problematic due to the scarcity of prokaryotic and eukaryotic expression systems which contain the protein processing machinery of eukaryotic secretory pathways [11,12]. Newly-developed approaches also suffer from limitations, such as cell-free protein expression, which allows for fast and efficient protein expression, yet suffers from inefficient folding and low protein activity [13,14]. Recombinant protein expression with different fusion tags has been successful in many cases, although it remains a challenge to express eukaryotic membrane proteins [1,7,9]. The human liver and lymph node sinusoidal endothelial cell C-type lectin (hLSECtin), a type II integral membrane protein, has a well-established biological activity, but the three-dimensional structure remains unknown due to low expression yields and aggregation into inclusion bodies [15-19].

The HIV-1 encoded trans-activator transcription (Tat) protein has been shown to deliver heterologous proteins across most biomembranes without losing their bioactivity [20-22]. The Tat core domain contains the sequence ‘YGRKKRRQRRR’ and it has been demonstrated that Tat peptide fusions can increase the yields and solubility of heterologous proteins [23]. Interestingly, our results here revealed that the Tat peptide was likely to promote the soluble expression of the membrane protein hLSECtin-CRD in *E. coli* without losing bioactivity and the protein expression level was not influenced compared with Tat-free proteins. It provided a novel method for the improvement of expression of soluble membrane proteins in prokaryotic cells by fusion with the Tat peptide, which may be potentially expanded to the expression of other membrane proteins.

## Materials and Methods

### Bacterial strains and plasmids

The bacterial strains BL21 (*E. coli* B F^–^ *dcm ompT hsd*S (r_B_^–^ m_B_^–^) *gal* λ) (DE3), the plasmids pET22b(+)-hLSECtin-CRD and pGEX-6P-hLSECtin-CRD were prepared in our laboratory. The plasmids pET28b and pET28b-Tat were provided by Dr. Chenggang Zhang from our institute. The plasmid pCDNA3.1a-myc-his-hLSECtin was provided by Dr. Li Tang from our institute.

### Construction of prokaryotic expression vectors

The hLSECtin-CRD cDNA fragments were amplified from pCDNA3.1a-myc-his-hLSECtin by PCR using two primers: pU: 5′-GCGGATCCGATGGGCTCCTGCTACTTTTTCTC-3′ (The *BamH*I restriction site is underlined), and pD: 5′-CCGCTCGAGGCAGTTGTGCCTTTTCTCACAGATC-3′ (The *Xho*I restriction site is underlined), and subcloned into the prokaryotic expression vectors pET28b and pET28b-Tat respectively. The prokaryotic expression vectors pET28b-Tat-hLSECtin-CRD and pET28b-hLSECtin-CRD were validated by direct sequencing.

### Expression of Tat-hLSECtin-CRD and hLSECtin-CRD

The above validated plasmids pET28b-Tat-hLSECtin-CRD and pET28b-hLSECtin-CRD were transformed into *E. coli* BL21 (DE3) cells and incubated at 37°C overnight. One clone from each plate was randomly picked and used to inoculate 5 mL LB medium containing kanamycin (final concentration: 200 μg/mL), followed by shaking at 37°C and 220 rpm until the logarithmic growth phase. The bacterial cells were diluted to an OD_600_ of 0.8 with fresh LB medium, then 2 mL of bacterial cells (approximately 1.3×10^8^ cells) were added to 100 mL LB media containing kanamycin (final concentration: 200 μg/mL) and shaken at 37°C and 220 rpm until the OD_600_ = 0.6–0.8. Subsequently, protein expression was induced with 1 mM IPTG at 20°C. Cells were harvested at 0, 2, 4 and 6 h, and subjected to ultrasonication in ice-cold PBS, and then centrifuged at 12,000 rpm for 10 min and filtered by a 0.45 μm filter. The protein expression level was identified subsequently using SDS-PAGE and Western blotting. All experiments were performed in triplicate.

### SDS-PAGE and Western blotting assay

Protein concentration was measured using the BCA protein assay reagent (Thermo Fisher Scientific Inc., USA) and equal mass (~35 μg) samples were fractionated by electrophoresis through 15% polyacrylamide gels. The gels were stained by Coomassie brilliant blue R-250 (Bio-Rad Laboratories, Inc., USA) and de-stained with a de-staining solution (10% acetic acid + 30% ethanol + 60% ddH_2_O). The images were gathered by Lane 1D image shooting software (Beijing SAGE Creation Science Co., Ltd, China).

Furthermore, equal mass samples (~35 μg) were also fractionated by electrophoresis through 15% polyacrylamide gels and transferred to polyvinylidene difluoride (PVDF) membranes (GE Healthcare) according to the manufacturer’s instructions. The membranes were probed with mouse-derived anti-6×His antibody (1:3,000 in TBST, Sigma, USA) and mouse-derived anti-GAPDH antibody (1:500 in TBST, Beijing Zhong-Shan Biotechnology, China) for 1.5 h at room temperature, followed with an HRP-conjugated goat anti-mouse secondary antibody (1:5,000 in TBST, Beijing Zhong-Shan Biotechnology, China) and incubated at room temperature for 1 h. After that, the chemiluminescent substrate luminal reagent (GE Healthcare, USA) and exposure to X-ray film were used to examine the immunolabeled bands. The optical density of the band was scanned and quantified with the ImageJ software version 1.46 (http://rsb.info.nih.gov/ij/, USA).

### Bacterial subcellular structure assay

The TEM ultramicrotomy assay was performed as previously reported [24]. The above-mentioned validated bacterial cells (approximately 50 mL), which overexpressed Tat-hLSECtin-CRD and hLSECtin-CRD proteins, were centrifuged at 5,000 rpm at room temperature for 10 min and washed with 1 mL ddH_2_O for three times. The bacterial cells were immobilized with 300 µL 2% Osmium tetroxide immobilization liquid for 2 days and sectioned using TEM ultramicrotomy (National Center of Biomedical Analysis, China). The bacterial subcellular structure images were captured using the AMT TEM system. All experiments were performed in triplicate.

### Mannose-binding activity experiment

To elucidate the binding activity of Tat-hLSECtin-CRD to mannose, based on the previously described method [25], the bacterial cells were split by ultrasonication in detergent extraction solution (150 mM NaCl, 10 mM Tris-HCl, pH 8.0, 0.15% Triton X-100, 1 mM MgCl_2_, and 10 mM CaCl_2_) and centrifuged at 12,000 rpm at 4°C for 10 min, after which the supernatant was collected. Subsequently, the supernatant (500 μL) was mixed with 500 μL loading buffer (150 mM NaCl, 25 mM Tris-HCl, pH 8.0, 25 mM CaCl_2_, 1 mM MgCl_2_ and 0.15% Triton X-100) and loaded onto a 1 mL mannose-agarose column (Sigma, USA). The flow-through was collected and columns were washed with 7 mL loading buffer followed with 7 mL elution buffer (150 mM NaCl, 25 mM Tris-HCl, pH 8.0, 0.15% Triton X-100, and 10 mM EDTA). The fractions were precipitated with 40 μg of bovine serum albumin (BSA) and 0.5 mL 30% trichloroacetic acid for 30 min on ice, and then centrifuged at 15,000 rpm at 4°C for 30 min. The pellets were washed twice with equal volumes of ethanol ether and dried at room temperature for 10 min, and then resuspended in 30 μL of reducing sample buffer. The equal volume samples were then prepared and fractionated by 15% SDS-PAGE and Western blot. All experiments were performed in triplicate.

### Statistical analysis

The above-mentioned data was statistically analyzed by the SPSS software (version 21.0, SPSS, USA, http://spss.en.softonic.com/) in groups compare using Student’s *t*-test with significant differences defined at *p* < 0.05, while *p* < 0.01 represents a highly significant difference.

## Results

### The constructed prokaryotic expression vectors

**Figure 1:**
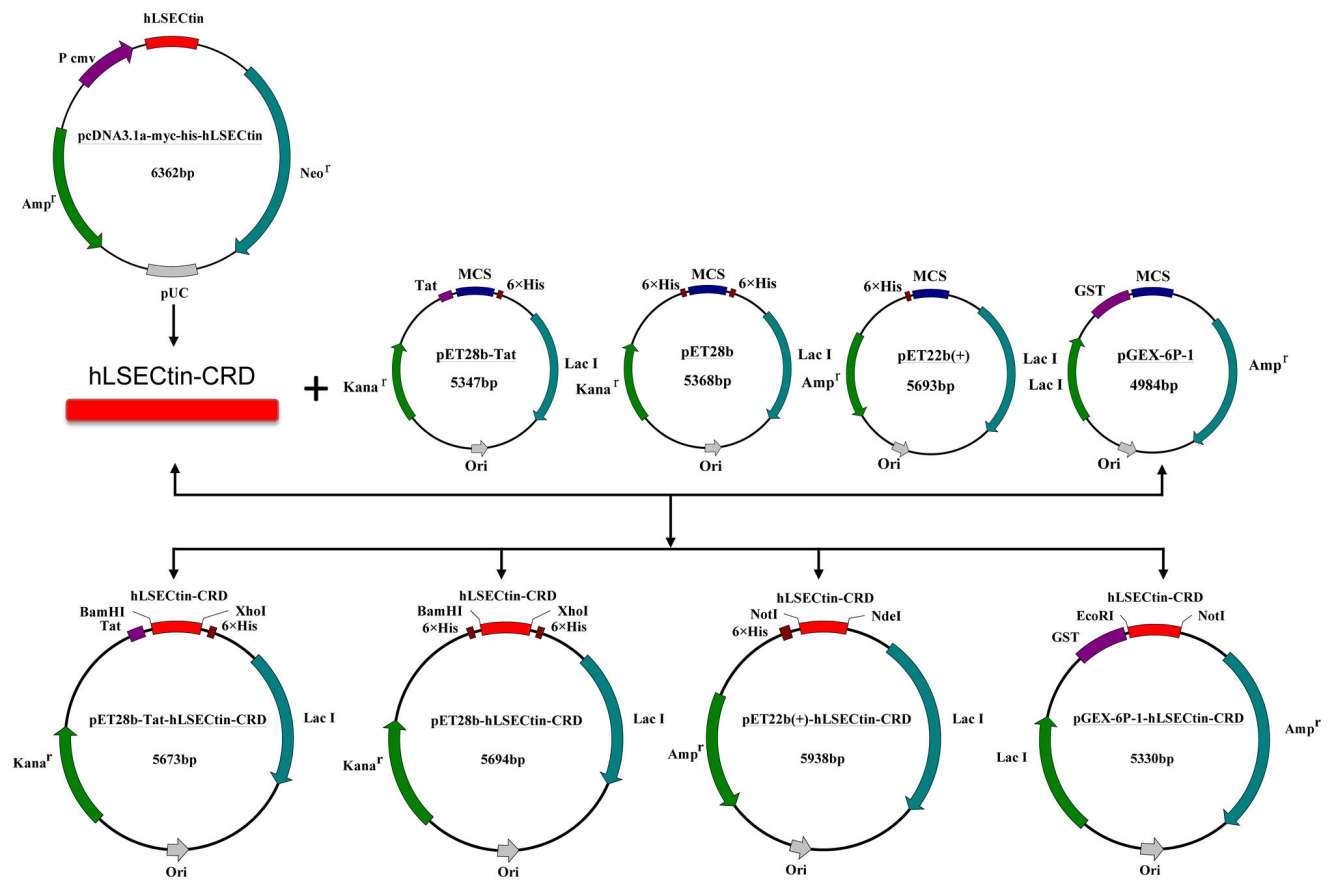
Schematic diagram showing the construction of the prokaryotic expression vectors. The hLSECtin-CRD (360bp) cDNA fragments were cloned into the prokaryotic expression vector pET28b-Tat, pET28b, pET22b (+) and pGEX-6P-1 to construct the prokaryotic expression vectors pET28b-Tat-hLSECtin-CRD, pET28b-hLSECtin-CRD, pET22b(+)-hLSECtin-CRD and pGEX-6P-1-hLSECtin-CRD.

The prokaryotic expression vector pET28b-Tat-hLSECtin-CRD (using pET28b-hLSECtin-CRD as a control) with the coding sequence of Tat in-frame was constructed and validated by direct sequencing. The prokaryotic expression vectors constructed in this study are illustrated in Table 1. The schematic diagram of prokaryotic vector construction is shown in Figure 1.

**Table 1:** Construction of the prokaryotic expression vectors used in this study.

Prokaryotic expression vectors	Complementary cDNA fragment	Size (bp)	Fusion protein	Protein size (kDa)
pET22b(+)-hLSECtin-CRD	*Nde*I-hLSECtin-CRD-*Not*I	393	hLSECtin-CRD-6×His	14.8
pGEX-6P-1-hLSECtin-CRD	*EcoR*I-hLSECtin-CRD-*Not*I	1062	GST-hLSECtin-CRD	40.8
pET28b-Tat-hLSECtin-CRD	*BamH*I-hLSECtin-CRD-*Xho*I	411	Tat-hLSECtin-CRD-6×His	16.4
pET28b- hLSECtin-CRD	*BamH*I-hLSECtin-CRD-*Xho*I	396	6×His-hLSECtin-CRD-6×His	15.7

### The Tat peptide promotes the soluble expression of the membrane protein hLSECtin-CRD in *E. coli*

**Figure 2:**
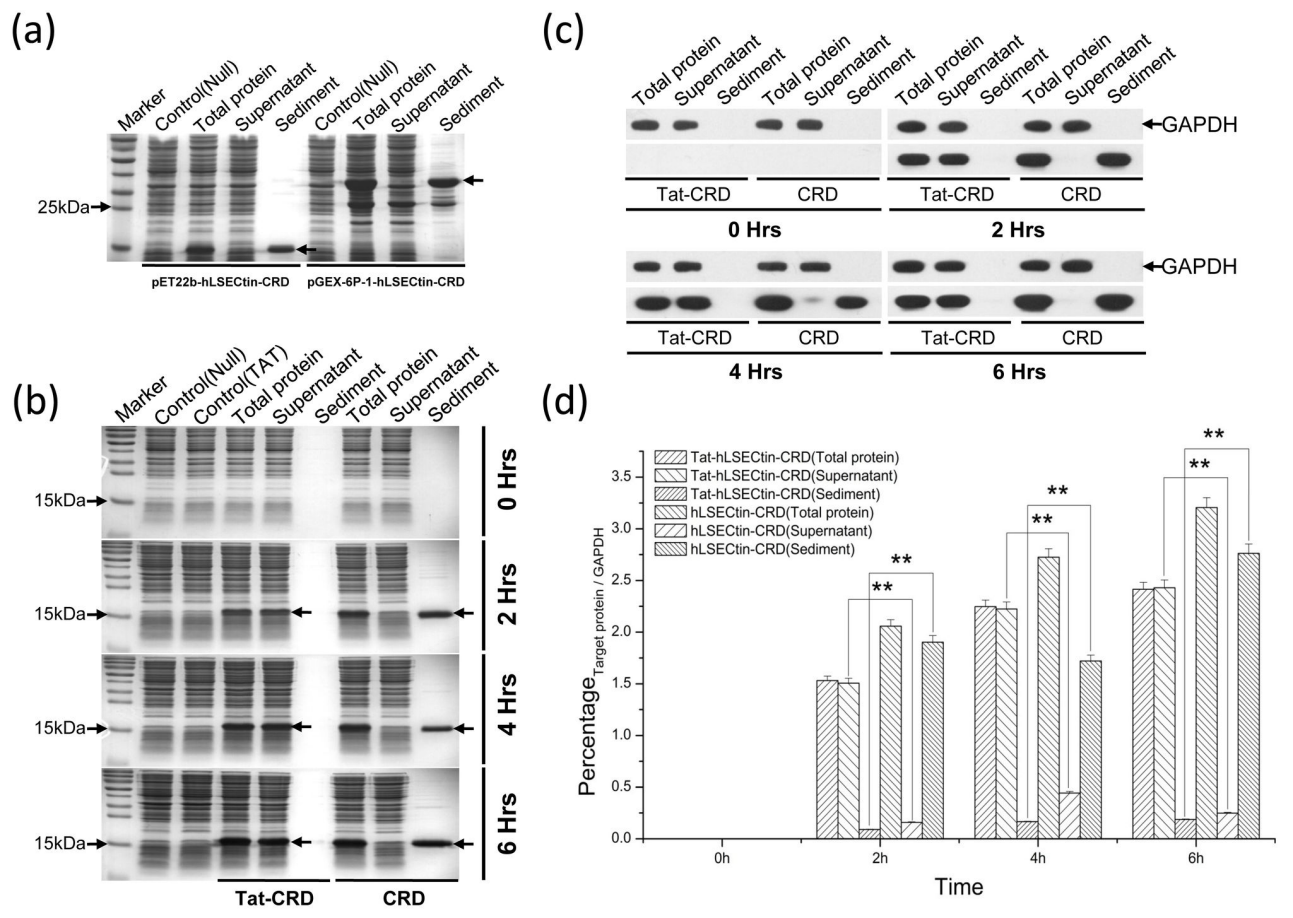
Identification of the protein expression level of Tat-hLSECtin-CRD and Tat-free hLSECtin-CRD at different time points by SDS-PAGE and Western blot with equal mass proteins, respectively. a: SDS-PAGE analysis of the total protein, the supernatant fraction and the sediment fraction of transformed *E. coli* with vectors pET22b(+)-hLSECtin-CRD, pGEX-6P-1-hLSECtin-CRD; b, c: SDS-PAGE and corresponding Western blot analysis of the total protein, the supernatant fraction and the sediment fraction respectively using GAPDH as a loading control at different time points; d: Histograms constructed from the Western blotting data show the difference in expression levels between Tat-hLSECtin-CRD and Tat-free hLSECtin-CRD expression fractions at different induction time points. ‘Control (Null)’ indicate the empty vector pET28b. ‘Control (Tat)’ indicate the empty vector pET28b-Tat. The arrowheads indicate target proteins. **: *p* < 0.01, as compared to Tat-free hLSECtin-CRD.

In our preceding work, the expression of the heterologous protein hLSECtin-CRD using the plasmids pET22b(+)-hLSECtin-CRD and pGEX-6P-1-hLSECtin-CRD in *E. coli* resulted in the protein being primarily aggregated as inclusion bodies (Figure 2a). To explore the feasibility of a Tat tag for the high-yield and soluble expression of hLSECtin-CRD, the Tat-tagged and Tat-free vectors (pET28b-Tat-hLSECtin-CRD and pET28b-hLSECtin-CRD) were constructed and equal mass expressed proteins were detected by SDS-PAGE (Figure 2b) and Western blot (Figure 2c and 2d) respectively. 

SDS-PAGE results demonstrated that Tat peptide did not influence the yields of Tat-hLSECtin-CRD, and could significantly increase its solubility. However, Tat-free proteins hLSECtin-CRD mainly expressed in the sediment (Figure 2b). Corresponding Western blotting assays confirmed the results (Figure 2c), and a subsequent histogram analysis showed a significant difference between Tat-tagged proteins (Tat-hLSECtin-CRD) and Tat-free proteins (hLSECtin-CRD) (Figure 2d, **p < 0.01). The Tat tag protein mainly expressed in the total protein and the supernatant, and not expressed in the sediment, but the Tat-free proteins mainly expressed in the total protein and the sediment, and not expressed in the supernatant. It indicated that Tat tag promoted soluble expression of heterologous proteins in *E. coli* and did not change the yields of heterologous proteins.

### The Tat tag facilitates the soluble expression of membrane proteins in *E. coli* BL21 (DE3) cells

**Figure 3:**
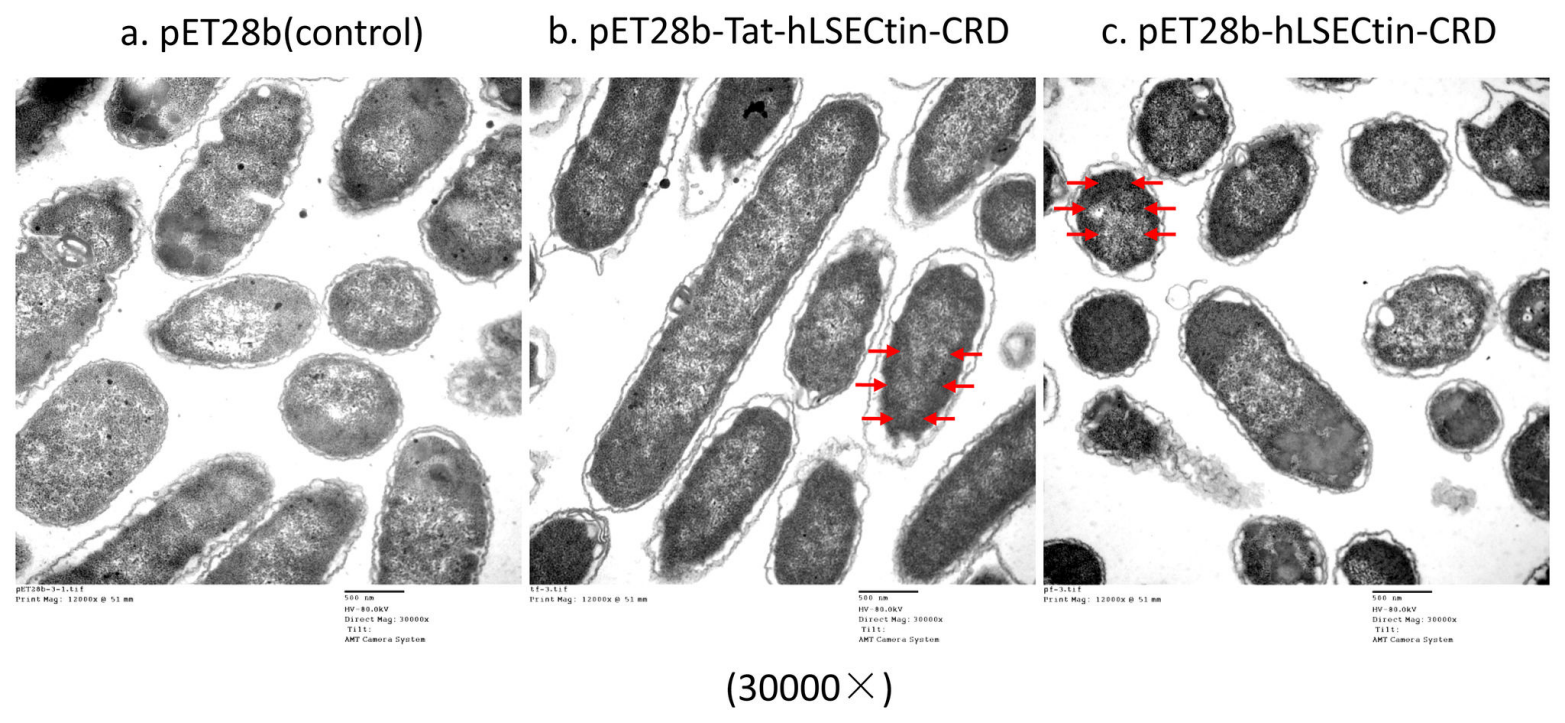
Identification of the subcellular microstructure of overexpressed Tat-hLSECtin-CRD or Tat-free hLSECtin-CRD bacterial cells by transmission electron microscopy. The arrowheads indicate different aggregation level of Tat-hLSECtin-CRD and Tat-free hLSECtin-CRD. a: pET28b(control); b: pET28b-Tat-hLSECtin-CRD, the red arrows indicate the denser region of Tat-hLSECtin-CRD in cytosol ; c: pET28b-hLSECtin-CRD, the red arrows indicate the dot denser region of Tat-free hLSECtin-CRD in cytosol.

The TEM ultramicrotomy as a routine technique has been widely applied in identifying of the inclusion body location in bacterial cells [24,26-29]. In this study, it was used to elucidate the subcellular structure changes of bacterial cells after overexpressed Tat tag and Tat free protein. The results demonstrated that the cytosol in bacterial cells which overexpressed Tat-hLSECtin-CRD and Tat-free hLSECtin-CRD proteins was denser than control cells (Figure 3a) as the red arrow displaying (Figure 3b and 3c), revealing that Tat-hLSECtin-CRD and Tat-free hLSECtin-CRD proteins have been overexpressed. In the bacterial cells expressing Tat-free hLSECtin-CRD proteins, the cytosol exhibited a dot dense area as the red arrow displaying (Figure 3c), indicating that the vast majority of Tat-free hLSECtin-CRD proteins aggregated as inclusion bodies. Simultaneously, the bacterial morphological structure containing Tat-hLSECtin-CRD (Figure 3b) was much more intact than the structure of cells containing Tat-free hLSECtin-CRD (Figure 3c). These results suggested that the Tat tag could facilitate the soluble expression of membrane proteins in *E. coli* BL21 (DE3) cells.

### Tat-hLSECtin-CRD possesses mannose-binding activity

**Figure 4:**
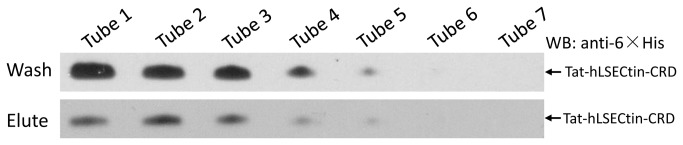
The mannose-binding activity of Tat-hLSECtin-CRD using mannose-agarose columns. The western blot showing the mannose-binding activity of washed and eluted fractions. A portion of Tat-hLSECtin-CRD proteins had bound on mannose-agarose column.

In previous studies, hLSECtin was proved to have carbohydrates-binding ability via its CRD domain, but this binding ability differed for various carbohydrates [18,25]. To elucidate the bioactivity of the Tat-tagged protein, the mannose-binding activity of Tat-hLSECtin-CRD was determined using mannose-agarose columns. These results demonstrated that Tat-hLSECtin-CRD can specifically bind to mannose, although the binding ability is weak (Figure 4), revealing that the Tat tag not only enhanced the solubility of membrane proteins but also does not disrupt their bioactivity and biological functions. Although the quantity of washed Tat-hLSECtin-CRD was greater than eluted Tat-hLSECtin-CRD, the mannose-binding activity of Tat-hLSECtin-CRD was confirmed.

## Discussion

Here, we showed that the HIV-1-encoded Tat peptide can increase the solubility of the membrane protein hLSECtin-CRD and the resulting fusion protein had mannose-binding activity. In addition, the bacterial subcellular structure of the cells which overexpressed Tat-hLSECtin-CRD exhibited denser region compared with controls, while dot denser region aggregated in bacterial cells after overexpressed Tat-free hLSECtin-CRD. Therefore, this study developed a novel method to express soluble membrane proteins using Tat fusions in *E. coli*, which is potentially applicable to other membrane proteins.

Membrane proteins, which account for approximately 20% to 30% of an organism’s proteome, play a key role in a variety of cellular functions, such as energy transformation, cell recognition and adhesion, signal transduction and material transportation [30-35]. Owing to their vital cellular functions, the study of membrane proteins has become the subject of intense research recently, especially the study of protein structure. Unfortunately, there is little structural information available for most membrane proteins due to the difficulties in obtaining sufficient bioactive protein [36]. Attempts at high-yield and soluble expression of membrane proteins have become a bottleneck in efforts to undertake structural or functional analyses [7,9]. Traditionally, eukaryotic membrane proteins were expressed in eukaryotic systems by recombinant techniques but with much lower yields [10]. The high-yield expression of these constructs could be achieved in prokaryotic systems but only for some prokaryotic proteins, since eukaryotic membrane proteins aggregated into inclusion bodies due to the lack of appropriate posttranslational modification mechanisms [11,12]. During the last decade, the advent of novel strategies to express membrane proteins has resulted in significant advancements such as baculovirus expression vector systems, cell-free expression systems, insect cells and mammalian cells expression systems *etc* [37,38]. However, no single method was suitable for all proteins. Previously, Wu et al. have shown that the HIV-1-encoded Tat peptide could increase the yields and the solubility of heterologous protein in prokaryotic expression systems [23]. Therefore, we hypothesized that the Tat peptide could also increase the yields and the solubility of the membrane protein hLSECtin-CRD.

LSECtin firstly cloned in the laboratory of our collaborator, contains Ca^2+^-dependent carbohydrate recognition domains (C-type CRDs) [18,39,40]. The biological activity of hLSECtin has been well documented, but little structural information exists due to the lack of abundant soluble protein for crystallization [41-44]. The gene is located on chromosome 19p13.3 and expressed in human peripheral blood and thymic dendritic cells, sinusoidal endothelial cells, and liver Kupffer cells [18,45]. As an endocytic receptor, an attachment factor and a glycan-binding receptor, hLSECtin could bind to various carbohydrates in a Ca^2+^-dependent manner via its CRD domain, including mannose, fucose and N-acetylglucosamine (GlcNAc) [46-48].In this study, we demonstrated that the Tat-hLSECtin-CRD protein has a distinct mannose-binding activity, suggesting that the Tat tag does not disrupt the bioactivity of heterologous proteins. Although our data effectively demonstrated that the Tat tag could promote the soluble expression of the membrane protein hLSECtin-CRD in *E. coli*, the mechanism is still unknown. The potential mechanism will be investigated in our future work that will focus on the regulation of gene expression and translation.

Overall, the current study documented that the Tat tag could promote the soluble expression of the membrane protein hLSECtin-CRD with inducer IPTG and without losing its bioactivity. As a novel method, the Tat tag technique could be applied to a variety of membrane proteins that are difficult to express in further structural studies.
